# Fibroblast-derived gremlin-1 Is associated with ovarian cancer-cell phenotypes in co-culture models

**DOI:** 10.3389/fonc.2026.1906132

**Published:** 2026-09-07

**Authors:** Haocheng Gao, Mengjuan Xia, Ting Yu, Xiaofang Chen, Xiaojuan Li, Heqiu Ruan

**Affiliations:** 1 Department of Gynecology, Hainan General Hospital/Hainan Medical University Hainan Hospital, Haikou, Hainan, China; 2 Medical Laboratory Center, Hainan General Hospital/Hainan Medical University Hainan Hospital, Haikou, Hainan, China; 3 Department of Pharmacy, Hainan General Hospital/Hainan Medical University Hainan Hospital, Haikou, Hainan, China; 4 Biomedical Statistics Office, Hainan General Hospital/Hainan Medical University Hainan Hospital, Haikou, Hainan, China

**Keywords:** cancer-associated fibroblasts, epithelial-mesenchymal Transition, FGFR1/MEK/ERK, grem1, ovarian cancer, tumor microenvironment

## Abstract

**Background:**

Cancer-associated fibroblasts (CAFs) can modify ovarian cancer-cell behavior through paracrine signals, but the contribution of CAF-derived Gremlin-1 remains incompletely defined.

**Methods:**

We compared stage IV and stage I ovarian-cancer transcriptomes in GSE9891, performed functional-enrichment analyses of differentially expressed genes, and interrogated CSIOVDB and KM Plotter for *GREM1* expression patterns, clinicopathologic associations, EMT-score correlation, and survival outcomes. We also measured *GREM1* mRNA in a small cohort of normal, benign and malignant ovarian tissues. *In vitro*, SKOV3 and A2780 cells were exposed to rGremlin-1, and GREM1 expression was manipulated by overexpression or knockdown in commercial fibroblasts marketed as CAFs (hereafter ‘CAFs’) under co-culture conditions. Cell viability, invasion, migration, apoptosis, EMT markers and, in SKOV3 cells, FGFR1/MEK/ERK phosphorylation were assessed.

**Results:**

GSE9891 DEGs were enriched in ECM-related processes. CSIOVDB associated higher *GREM1* expression with tumor stroma, peritoneal metastasis, advanced stage, higher grade, a mesenchymal subtype, and EMT score. In a small exploratory tissue cohort, *GREM1* expression showed a numerical increase in benign and malignant ovarian tumors relative to normal tissue, consistent in direction with the CSIOVDB findings, but this pattern was not statistically significant and should be interpreted as descriptive rather than confirmatory. rGremlin-1 exposure altered invasion, migration, EMT markers, and apoptosis, while the SKOV3 CCK-8 response was biphasic. CAF *GREM1* gain- and loss-of-function produced opposing changes in several co-culture phenotypes, although some responses were cell-line or time dependent. In SKOV3 cells, OE GREM1 CAFs were associated with higher p-FGFR1/FGFR1, p-MEK1/2/MEK1/2 and p-ERK1/2/ERK1/2 ratios; shGREM1 reduced the p-ERK1/2/ERK1/2 ratio, whereas the p-FGFR1/FGFR1 and p-MEK1/2/MEK1/2 contrasts were not significant.

**Conclusion:**

CAF *GREM1* expression was associated with selected phenotypes in SKOV3 and A2780 cells under the tested *in vitro* conditions. FGFR1/MEK/ERK phosphorylation patterns in SKOV3 cells suggested a candidate signaling link. Further validation in independently characterized CAF models and additional ovarian-cancer systems is needed.

## 1 Introduction

Ovarian cancer (OC) remains a major cause of gynecologic-cancer mortality, and outcomes are particularly poor when disease presents at an advanced stage or recurs after platinum-based therapy (1). Although surgery and systemic treatment improve outcomes for some patients, residual disease and treatment resistance continue to limit durable control (2). Due to the lack of reliable early detection methods, most OC patients are diagnosed at an advanced stage (Stage III/IV), making treatment exceedingly difficult and resulting in a five-year survival rate of less than 30% (3–5). The current standard of care consists of debulking surgery followed by platinum-based chemotherapy. However, residual post-surgical lesions, along with primary and acquired platinum resistance, severely limit long-term survival (6, 7). Therefore, identifying novel molecular targets to overcome drug resistance and impede tumor progression is of paramount importance.

Strategies targeting the tumor microenvironment (TME) have emerged as promising approaches to suppress TME-mediated resistance and improve clinical outcomes. Tumor-stroma crosstalk, as a key focus of these strategies, is mediated by growth factors, cytokines, chemokines, exosomes and extracellular-matrix signals that can reshape tumour-cell behaviour and treatment response (8, 9). Cancer-associated fibroblasts (CAFs) are abundant and heterogeneous stromal cells that contribute to matrix remodelling, invasion, epithelial-mesenchymal plasticity, therapy resistance, angiogenesis and drug resistance (10–12). In ovarian cancer, however, the individual paracrine factors that account for these effects remain incompletely resolved.

Gremlin-1, encoded by the *GREM1* gene, is a secreted antagonist of bone morphogenetic protein (BMP) signalling (13, 14). By binding BMP-2, BMP-4 and BMP-7, Gremlin-1 can limit ligand-receptor engagement and alter downstream signalling (15). Studies in other tumour types have implicated Gremlin-1 in stromal remodelling, lineage plasticity, metastasis and therapy-relevant phenotypes (16). These observations support Gremlin-1 as a candidate stromal signal, but they do not establish how CAF-associated Gremlin-1 relates to ovarian-cancer-cell behaviour. The functional effects of secreted Gremlin-1 are likely to be context dependent, and the specific contribution of CAF-derived Gremlin-1 in ovarian cancer remains insufficiently defined (17–20).

Here, we investigated this question using complementary association and perturbation approaches. We examined advanced-stage transcriptional changes and public ovarian-cancer expression datasets, measured *GREM1* mRNA in an independent small tissue cohort, and tested recombinant human Gremlin-1 (rGremlin-1) in SKOV3 and A2780 cells. We also manipulated *GREM1* in commercially supplied fibroblasts marketed as CAFs before co-culture. We assessed CCK-8 signal, invasion, wound closure, apoptosis and EMT-marker changes; in SKOV3 cells, we additionally examined FGFR1/MEK/ERK phosphorylation as a candidate signalling correlate. This design was intended to detect candidate associations between CAF *GREM1* status and malignant phenotypes in the tested *in vitro* models.

## 2 Materials and methods

### 2.1 Identification of differentially expressed genes

The GSE9891 microarray dataset was obtained from the Gene Expression Omnibus (GEO) database and analyzed on the GPL570 Affymetrix Human Genome U133 Plus 2.0 platform (21, 22). The GEO2R tool (https://www.ncbi.nlm.nih.gov/geo/geo2r/) and the limma workflow were used to compare FIGO stage IV with FIGO stage I OC samples. Differentially expressed genes (DEGs) were defined as |log2 fold-change (FC)| ≥ 1.5 and adjusted *P*-value < 0.05 (Benjamini-Hochberg false discovery rate, FDR).

### 2.2 Gene ontology and pathway-enrichment analysis

Enrichr (https://maayanlab.cloud/enrichr/) was used for Gene Ontology and pathway-enrichment analysis of the DEGs (23). Biological Process, Cellular Component, and Molecular Function terms were examined together with Reactome, BioPlanet, and WikiPathways collections. Enrichment terms were ranked using the Enrichr combined score; nominal *P* < 0.05 and *Q* < 0.25 were used to describe the reported enrichment output.

### 2.3 CSIOVDB expression and correlation analyses

**Figure 1:**
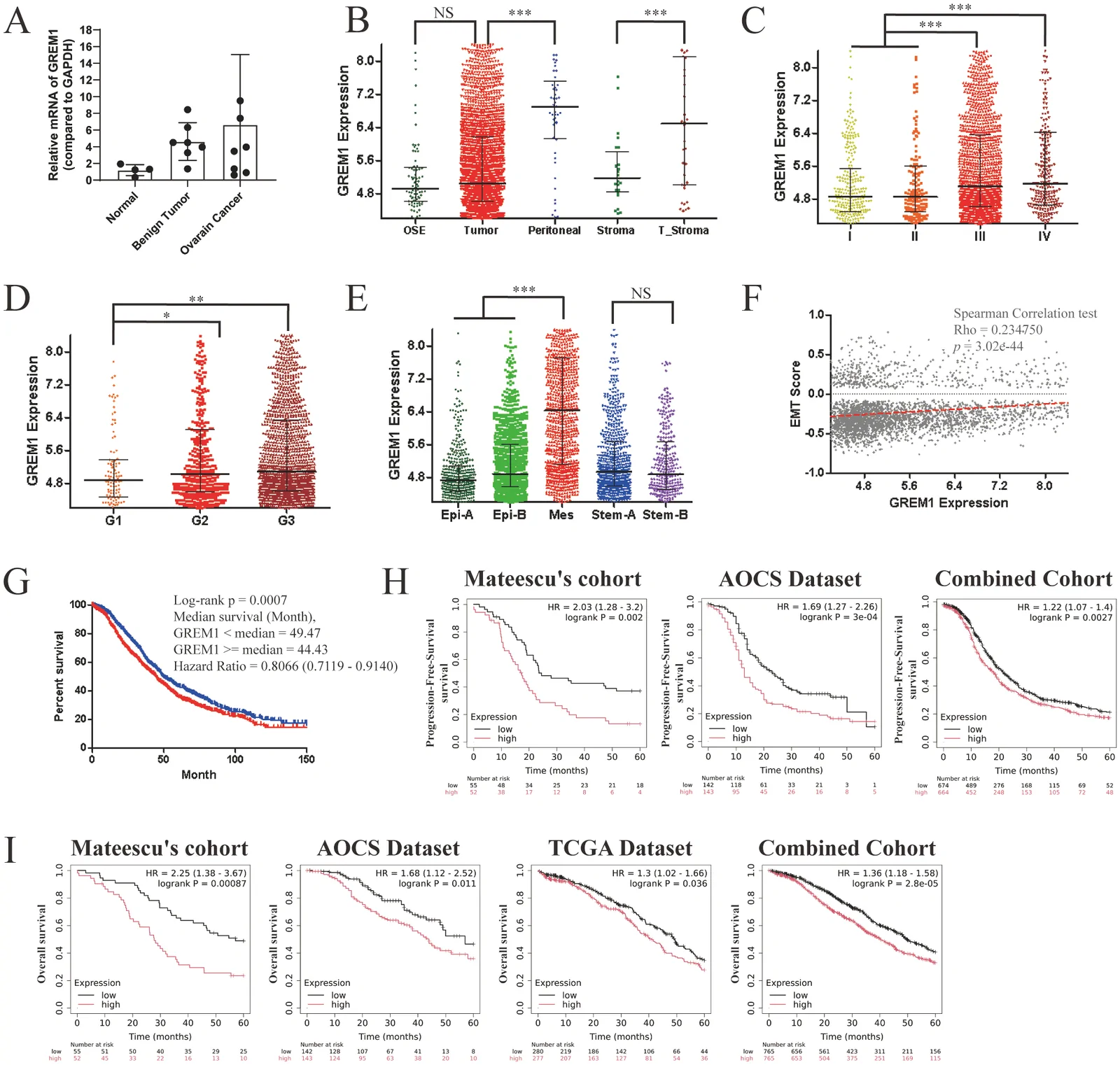
*GREM1* expression and clinical associations in ovarian cancer. **(A)**Relative *GREM1* mRNA abundance in normal ovarian tissue (*n* = 4), benign ovarian tumors (*n* = 7) and malignant ovarian tumors (*n* = 8), measured by qRT-PCR. Data are presented as mean ± SD. The overall comparison was performed using the exact Kruskal-Wallis test followed by Dunn’s multiple-comparisons test. The overall difference was not significant (*H* = 5.118, *P* = 0.0720), and no adjusted pairwise comparison was significant. **(B)** *GREM1* expression in normal ovarian surface epithelium (OSE), primary ovarian tumors, peritoneal metastases, normal ovarian stroma and ovarian tumor stroma (T-stroma) in the CSIOVDB database. Asterisks indicate the comparisons shown in the panel. **(C)** *GREM1* expression in ovarian cancer specimens grouped by FIGO stage. Asterisks indicate comparisons with stage I/II. **(D)** *GREM1* expression in specimens grouped by histological grade. Asterisks indicate comparisons with grade 1. **(E)** *GREM1* expression across ovarian cancer molecular subtypes (Epi-A, Epi-B, Mes, Stem-A and Stem-B). Asterisks indicate comparisons with the Epi-A/Epi-B subtypes. **(F)** Correlation between *GREM1* expression and EMT scores in the CSIOVDB database. Spearman’s ρ = 0.235, *P* = 3.02 × 10^-44^. **(G)** Kaplan-Meier analysis of overall survival (OS) in the CSIOVDB database according to *GREM1* expression. **(H)** Kaplan-Meier analysis of progression-free survival (PFS) in the Mateescu cohort (GSE26193, *n* = 107), AOCS (GSE9891, *n* = 285) and the combined cohort (*n* = 1,338) from the KM Plotter database. **(I)** Kaplan-Meier analysis of OS in the Mateescu cohort (GSE26193, *n* = 107), AOCS (GSE9891, *n* = 285), TCGA dataset (*n* = 557) and the combined cohort (*n* = 1,530) from the KM Plotter database. The median *GREM1* expression was used to define the low- and high-expression groups. Hazard ratios with 95% confidence intervals and log-rank *P* values are shown in each survival plot. *P < 0.05, **P < 0.01 and ***P < 0.001; ns, not significant. AOCS, Australian Ovarian Cancer Study; EMT, epithelial-to-mesenchymal transition; FIGO, International Federation of Gynecology and Obstetrics; OSE, ovarian surface epithelium; OS, overall survival; PFS, progression-free survival; TCGA, The Cancer Genome Atlas.

*GREM1* expression was examined in CSIOVDB, a public ovarian-cancer expression database with clinicopathologic annotations (24, 25). Group comparisons across tissue compartments, FIGO stages, histologic grades, and molecular subtypes are shown in **Figures 1B–E**, with significance annotations transcribed from the corresponding CSIOVDB outputs. The association between *GREM1* expression and the database EMT score was evaluated using Spearman correlation and is shown in **Figure 1F**. Survival curves based on *GREM1* expression in the CSIOVDB cohort are shown in **Figure 1G** and were compared using Kaplan-Meier analysis and the log-rank test.

### 2.4 Cell lines, fibroblasts, and recombinant protein

SKOV3 and A2780 cells were obtained from Wuhan Procell Life Science & Technology Co., Ltd. (Wuhan, China) and maintained in McCoy’s 5A medium supplemented with 10% fetal bovine serum, 100 U/mL penicillin, and 100 µg/mL streptomycin at 37 °C in 5% CO_2_. Cell-line identity was assessed by 21-locus short tandem repeat profiling. The A2780 sample showed a basic match to the A2780 reference profile in the ExPASy database (EV = 0.94; CVCL_0134), whereas the SKOV3 sample showed an exact match to the SK-OV-3 reference profile in the DSMZ database (EV = 1.00; HPACC).

Commercial fibroblasts marketed by the supplier as CAFs were obtained from ICell Bioscience (Shanghai, China) and maintained in the ICell Primary Fibroblast Culture System under the same temperature and CO_2_ conditions. The preparation was used as supplied and was authenticated by the supplier. Vimentin immunofluorescence showed more than 90% Vimentin-positive cells (**Supplementary Figure S1**), supporting their fibroblast lineage. However, because Vimentin is a general fibroblast marker rather than a CAF-specific marker, these data do not establish a complete CAF phenotype. Hereafter, these fibroblasts are referred to as CAFs for brevity.

Recombinant human Gremlin-1 (rGremlin-1; CSB-YP009892HU, Cusabio, Wuhan, China; stated purity ≥85% by SDS-PAGE) was reconstituted in sterile deionized water to 100 µg/mL, supplemented with 10% glycerol, aliquoted and stored at -80 °C. Working solutions were prepared by dilution in culture medium. The manufacturer’s certificate of analysis reported an endotoxin level below 1 EU/µg. No neutralizing-antibody, heat-denatured-protein or matched-protein control was available; therefore, the recombinant-protein experiments were interpreted as supportive evidence rather than definitive evidence of ligand-specific effects.

### 2.5 Cell co-culture

CAFs (as defined in Section 2.4) and OC cells were co-cultured in Transwell systems with 0.4-µm pores (Corning, NY, USA) at a CAF-to-cancer-cell ratio of 1:2. CAFs were seeded in the lower chamber and SKOV3 or A2780 cells in the upper chamber. Both compartments contained a 1:1 mixture of McCoy’s 5A medium and ICell Primary Fibroblast Culture System supplemented with 10% fetal bovine serum and antibiotics. Unless otherwise stated, co-culture was maintained for 48 h before downstream measurements.

### 2.6 Patient samples

The study was approved by the Ethics Committee of Hainan General Hospital and was conducted in accordance with the Declaration of Helsinki. Written informed consent was obtained from all participants. The tissue cohort comprised normal ovarian tissue (*n* = 4), benign ovarian tumor tissue (*n* = 7), and malignant ovarian tumor tissue (*n* = 8). Diagnoses were established by routine pathology. Tissue samples were snap-frozen in liquid nitrogen and stored at -80 °C.

RNA was extracted from bulk whole-tissue homogenates without macrodissection, microdissection, stromal enrichment, or CAF isolation. These qRT-PCR measurements therefore represent tissue-level *GREM1* expression and do not establish cell- or compartment-specific localization.

### 2.7 Lentiviral gain- and loss-of-function in CAFs

Lentiviral constructs were generated and packaged by Shanghai GeneChem Co., Ltd. (Shanghai, China). Recombinant GV vectors were packaged in 293T cells by co-transfection with pHelper 1.0 and pHelper 2.0, concentrated by ultracentrifugation, and titrated by quantitative PCR. Supplier-reported titers were 1-2 × 10^9^ TU/mL for the shRNA lentiviruses and 2.5 × 10^8^ TU/mL for the *GREM1* overexpression lentivirus. According to the supplier’s quality-control report, the viral stocks were negative for mycoplasma, Chlamydia, bacterial and fungal contamination, with endotoxin levels below 50 EU/mL.

Three shRNAs targeting *GREM1* were used: shGREM1-1, 5′-CAGGCTGATGCTTCTGAGAAA-3′; shGREM1-2, 5′-AGGGACATTGCAGAAGCTTGA-3′; and shGREM1-3, 5′-TAGCCAAGTCCTATGTAATAT-3′. The non-targeting control was shNC, 5′-TTCTCCGAACGTGTCACGT-3′. The full-length human *GREM1* coding sequence (NM_013372) was cloned into the GV492 lentiviral vector for overexpression.

CAFs were infected at a multiplicity of infection of 50 and selected with 3 µg/mL puromycin for 7 days. The resulting conditions were designated OE NC, OE GREM1, shNC, and shGREM1. *GREM1* expression was verified by qRT-PCR and western blotting. Because shGREM1–2 produced the strongest knockdown, it was used in subsequent experiments and is referred to as shGREM1 in the figures.

### 2.8 OC survival analysis in datasets

Kaplan-Meier survival analyses were conducted using the KM Plotter online database (www.kmplot.com). For progression-free survival (PFS) analysis, multiple independent cohorts were included (the AOCS [GSE9891, *n* = 285], Mateescu’s cohort [GSE26193, *n* = 107], and a combined cohort [*n* = 1,338] of all available datasets). For overall survival (OS) analysis, the same cohorts, together with the TCGA dataset [*n* = 557], were analyzed. Patients in all cohorts ranged from FIGO stage I to IV. *P* values were calculated using the log-rank (Mantel-Cox) test. Patients were stratified into “low” and “high “ expression groups based on the median expression level as the cutoff.

### 2.9 Quantitative reverse-transcription

Total RNA was extracted from bulk ovarian-tissue homogenates or cultured cells using TRIzol reagent (15596-026; Invitrogen, Carlsbad, CA, USA). Reverse transcription was performed using the cDNA Reverse Transcription Kit (11141ES60; Yeasen Biotechnology, Shanghai, China). GAPDH served as the reference gene, and relative *GREM1* mRNA abundance was calculated using the 2^−ΔΔCt^ method. Primer sequences are provided in **Table 1**.

**Table 1:** Primer sequences used for qRT-PCR.

Gene	Primer sequences (5′-3′)
*GREM1*	Forward: ACAAGGCCCAGCACAAT Reverse: GCACCAGTCTCGCTTCA
*GAPDH*	Forward: CCTTCCGTGTCCCCACT Reverse: GCCTGCTTCACCACCTTC

*GREM1*, gremlin 1; *GAPDH*, glyceraldehyde-3-phosphate dehydrogenase.

### 2.10 Western blotting

Cells were lysed in RIPA buffer (Beyotime Biotechnology, Shanghai, China), and protein concentrations were measured by bicinchoninic acid assay (P0010; Beyotime Biotechnology, Shanghai, China). Equal protein amounts (25 µg per lane) were separated by SDS-PAGE and transferred to PVDF membranes. Membranes were blocked in 5% non-fat milk/TBST for 1 h and incubated overnight at 4 °C with antibodies against GREM1 (K008160P; Solarbio; 1:500), E-cadherin (AF0131; Affinity Biosciences; 1:1,000), Vimentin (AF7013; Affinity Biosciences; 1:1,000), N-cadherin (AF5239; Affinity Biosciences; 1:1,000), beta-actin (66009-1-Ig; Proteintech; 1:20,000), p-FGFR1 (BS-13155R; Bioss; 1:500), FGFR1 (YP-AB-17298; UpingBio; 1:500), p-MEK1/2 (YP-AB-17778; UpingBio; 1:500), MEK1/2 (YP-AB-04464; UpingBio; 1:500), p-ERK1/2 (BS-3016R; Bioss; 1:500), and ERK1/2 (YP-AB-04513; UpingBio; 1:1,000). HRP-conjugated secondary antibodies were applied for 1 h at room temperature. Signals were visualized by enhanced chemiluminescence and quantified in ImageJ version 1.8.0. Target proteins were normalized to β-actin; phosphorylation outcomes were expressed relative to the corresponding total protein.

### 2.11 Wound healing assay

Cells were grown to confluence in 6-well plates. A linear wound was generated with a sterile 200-µL pipette tip, detached cells were removed with PBS, and cultures were maintained in serum-free medium. The same wells were imaged at 0, 24, and 48 h. Wound width was measured in ImageJ, and relative migration distance was calculated as (W_0_ - W_t_)/W0 × 100%, where W_0_ is the width at 0 h and W_t_ is the width at the indicated follow-up time. Because the same wells were followed longitudinally, time-course inference used repeated-measures models.

### 2.12 Transwell invasion assay

Invasion was assessed using Matrigel-coated 8-µm Transwell inserts (3422; Corning). For recombinant-protein experiments, cancer cells were suspended in serum-free medium and seeded in the upper chamber, whereas the lower chamber contained medium supplemented with 10% fetal bovine serum and the indicated concentration of rGremlin-1. For CAF co-culture experiments, treatment-matched CAFs were seeded in the lower chamber and cancer cells in the upper chamber, with both compartments maintained in the co-culture medium described in Section 2.5. After 24 h, non-invading cells were removed, and invaded cells were fixed with 4% paraformaldehyde and stained with 0.1% crystal violet. Three randomly selected microscopic fields were counted per insert at 200 × magnification. Field counts were averaged within each independent experiment before inferential analysis; fields were not treated as independent replicates.

### 2.13 Flow cytometric analysis

Apoptosis was measured using an Annexin V-FITC/PI Apoptosis Detection Kit (40302ES50; Yeasen Biotechnology, Shanghai, China). Stained cells were analyzed on a CytoFLEX S flow cytometer (Beckman Coulter, Brea, CA, USA), and data were processed in CytExpert (Beckman Coulter, Brea, CA, USA). The apoptosis outcome used for analysis was derived separately from each independent experiment.

### 2.14 CCK-8 cell viability assay

Cells were seeded at 3 × 10^3^ cells per well in 96-well plates. After the indicated treatment or co-culture condition, 10 µL of Cell Counting Kit-8 reagent (Beijing LABGIC Technology Co., Ltd.) was added for 2 h at 37 °C, and absorbance was measured at 450 nm by using a microplate reader (Multiskan MK3; Thermo Fisher Scientific, Waltham, MA, USA). Technical wells were averaged within each independent experiment before analysis.

### 2.15 Statistical analysis

Statistical analyses were performed using GraphPad Prism 10. Data are presented as mean ± SD unless otherwise indicated. *n* denotes the number of independent biological experiments; technical replicates, microscopic fields, and repeated measurements were averaged within each experiment and were not treated as independent observations. No data points were excluded. One-way ANOVA was used for single-factor comparisons. Two-way ANOVA was used when two experimental factors were evaluated simultaneously, including their interaction. Repeated-measures two-way ANOVA was used for longitudinal data with repeated observations from the same experimental units; the Geisser-Greenhouse correction was applied when sphericity was not assumed. Nonparametric outcomes were analyzed using the Kruskal-Wallis test. Dunnett’s test was used for prespecified comparisons with a common control, Tukey’s test for all-pairwise comparisons, and Dunn’s test following Kruskal-Wallis analyses. Selected outcomes were log-transformed before analysis when appropriate. Multiplicity-adjusted *P* values are reported where applicable. All tests were two-sided, and *P* < 0.05 was considered statistically significant. All cell-based assays were performed in three independent biological experiments (*n* = 3). We acknowledge that this replication level provides limited statistical power to detect modest but biologically meaningful effects; non-significant outcomes are therefore interpreted as inconclusive rather than as evidence of absence of an effect, and test statistics are reported alongside *P* values where applicable.

## 3 Results

### 3.1 Identification of DEGs associated with advanced-stage OC and enrichment analysis

**Figure 2:**
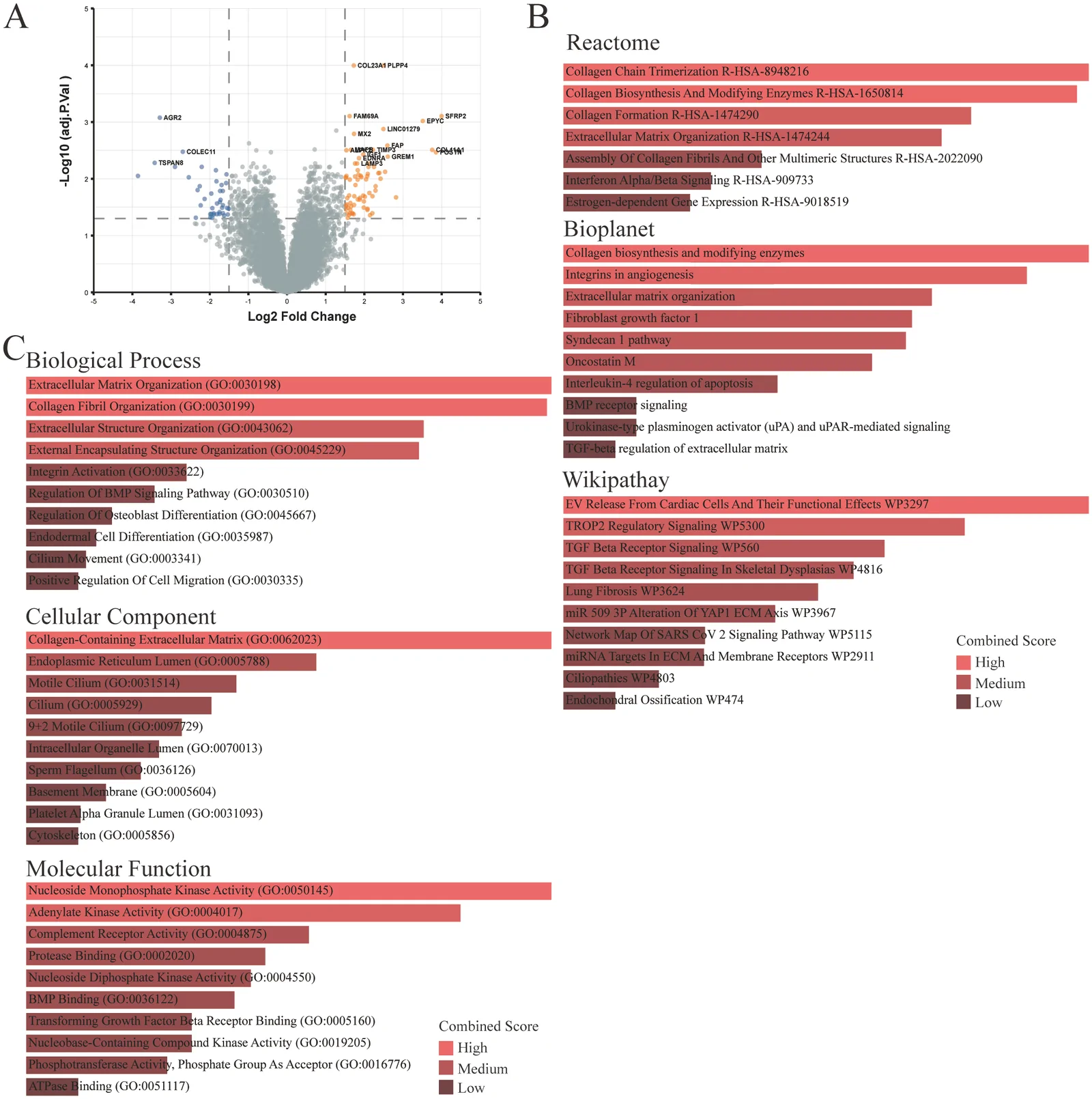
Differentially expressed genes and pathway enrichment associated with advanced-stage ovarian cancer. **(A)** Volcano plot comparing FIGO stage IV and stage I ovarian tumors in GSE9891. Genes meeting the prespecified FDR and fold-change thresholds are highlighted. A total of 113 differentially expressed genes (DEGs) were identified, including 73 upregulated and 40 downregulated genes in stage IV tumors. **(B)** Enrichment analysis of the DEGs using Reactome, BioPlanet and WikiPathways. Representative enriched terms included collagen biosynthesis and fibril assembly, extracellular matrix (ECM) organization, integrin and syndecan signaling, fibroblast growth factor 1, uPA/uPAR-mediated signaling, and BMP receptor and TGF-β signaling pathways. **(C)** Gene Ontology enrichment analysis of biological process, cellular component and molecular function terms. Enriched terms emphasized ECM organization, collagen fibril organization, collagen-containing ECM and related molecular functions. DEGs, differentially expressed genes; ECM, extracellular matrix; FIGO, International Federation of Gynecology and Obstetrics; FDR, false-discovery rate; OC, ovarian cancer.

To characterize transcriptional differences associated with advanced-stage disease, we compared FIGO stage IV and stage I tumors in GSE9891. The comparison identified 113 DEGs using the prespecified FDR and fold-change thresholds, including 73 upregulated and 40 downregulated genes in stage IV tumors (**Figure 2A**). Across the Reactome, BioPlanet and WikiPathways analyses, enriched terms converged on collagen biosynthesis and fibril assembly, extracellular matrix (ECM) organization, integrin and syndecan signaling, fibroblast growth factor 1, uPA/uPAR-mediated signaling, and BMP receptor and TGF-β signaling pathways (**Figure 2B**). Gene Ontology Biological Process and Cellular Component analyses similarly emphasized ECM organization, collagen fibril organization and collagen-containing ECM (**Figure 2C**). Together, these enrichment analyses indicated that the stage IV-associated transcriptional profile was enriched for ECM organization, collagen-related biology and associated signaling pathways.

### 3.2 *GREM1* expression and retrospective clinical associations in ovarian cancer

Analysis of 3,431 OC samples in CSIOVDB (24) showed higher *GREM1* expression in ovarian tumor stroma than in normal ovarian stroma and higher expression in peritoneal metastases than in primary tumors (both *P* < 0.001; **Figure 1B**). *GREM1* expression was also higher in advanced FIGO stages (*P* < 0.001; **Figure 1C**), in higher-grade tumors (*P* < 0.05; **Figure 1D**), and in the mesenchymal molecular subtype (25) relative to the epithelial Epi-A and Epi-B subtypes (*P* < 0.001; **Figure 1E**). *GREM1* expression was positively correlated with the EMT score (Spearman’s ρ = 0.235, *P* = 3.02 × 10^-44^; **Figure 1F**).

In an independent exploratory tissue cohort, mean relative *GREM1* mRNA values were 1.19 ± 0.66 in normal ovarian tissue (*n* = 4), 4.62 ± 2.25 in benign ovarian tumors (*n* = 7), and 6.63 ± 8.40 in malignant ovarian tumors (*n* = 8). Values were numerically higher in both tumor groups than in normal tissue; however, the overall difference was not significant (exact Kruskal-Wallis test, H = 5.118, *P* = 0.0720), and no pairwise comparison remained significant after Dunn’s adjustment (all adjusted *P* > 0.05). This directional pattern was consistent with higher *GREM1* expression in tumor-associated stroma in CSIOVDB. The small cohort and use of bulk-tissue RNA may have reduced sensitivity to stromal-specific differences.

Survival analyses across multiple cohorts consistently associated high *GREM1* expression with poorer outcomes. In the CSIOVDB dataset, high *GREM1* expression correlated with shorter OS (**Figure 1G**). Similar associations for OS (**Figure 1I**; all *P* < 0.05) were observed in the AOCS (GSE9891, *n* = 285), Mateescu’s cohort (GSE26193, *n* = 107), TCGA dataset (*n* = 557), and the combined cohort (*n* = 1,530) from the KM Plotter database. The same trend was observed for PFS in the AOCS, Mateescu’s cohort, and the combined cohort (**Figure 1H**; all *P* < 0.05). Collectively, these analyses associated higher *GREM1* expression with tumor-associated stromal and metastatic contexts and poorer survival.

### 3.3 Recombinant gremlin-1 alters ovarian cancer-cells phenotypes

**Figure 3:**
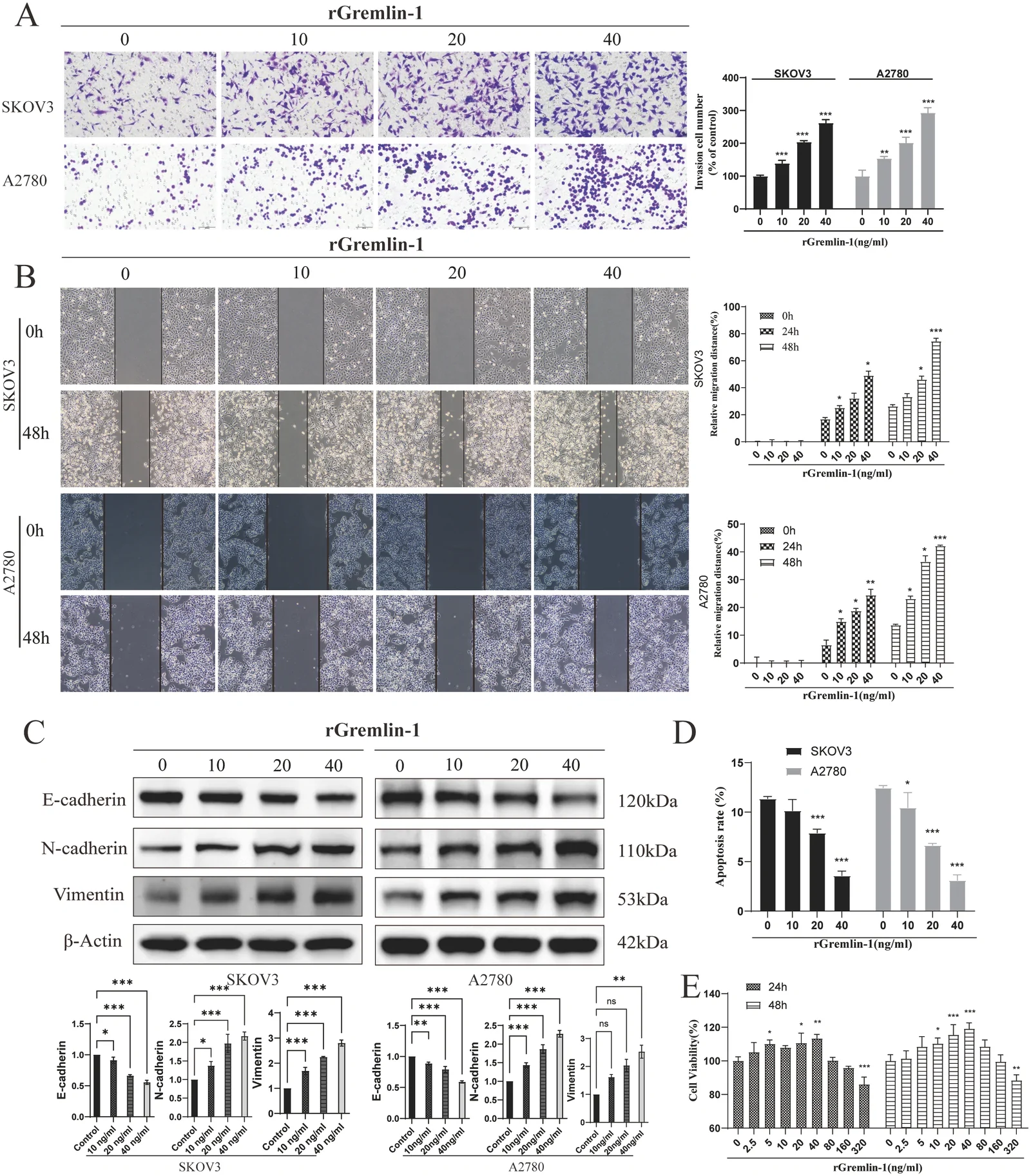
Recombinant Gremlin-1 alters ovarian cancer-cell phenotypes. **(A)** Transwell invasion assay of SKOV3 and A2780 cells treated with recombinant human Gremlin-1 (rGremlin-1; 10, 20 or 40 ng/mL) for 24 h. Representative images are shown at ×200 magnification. **(B)** Wound-healing assay of SKOV3 and A2780 cells treated with rGremlin-1 (10, 20 or 40 ng/mL) and monitored at 0, 24 and 48 h. Representative images at 0 and 48 h are shown at ×200 magnification. Longitudinal measurements were analyzed using repeated-measures two-way ANOVA with Dunnett-adjusted simple-effects comparisons. **(C)** Western blot analysis of E-cadherin, N-cadherin and Vimentin in SKOV3 and A2780 cells treated with rGremlin-1 for 24 h. β-actin served as the loading control. **(D)** Flow-cytometric analysis of apoptosis using Annexin V-FITC/PI staining in SKOV3 and A2780 cells treated with rGremlin-1 for 24 h. **(E)** CCK-8 assay of SKOV3 cells treated with rGremlin-1 at concentrations ranging from 2.5 to 320 ng/mL for 24 or 48 h. Data are presented as mean ± SD (*n* = 3 independent experiments). One-way ANOVA with Dunnett’s multiple-comparisons test was used for panels **(A, C, D)**, except for A2780 Vimentin in panel **(C)**, which was analyzed using the exact Kruskal-Wallis test followed by Dunn’s test. Panel **(E)** was analyzed using two-way ANOVA with Dunnett-adjusted comparisons. *P < 0.05, **P < 0.01 and ***P < 0.001 versus the control group.

To examine the phenotypic effects of exogenous Gremlin-1 in ovarian-cancer cells, we assessed invasion, migration, apoptosis and EMT-related markers. In SKOV3 cells, rGremlin-1 (2.5–320 ng/mL) produced significant main effects of concentration (*F*(8,36) = 29.43, *P* < 0.001) and time (*F*(1,36) = 5.123, *P* < 0.05), without a concentration-by-time interaction (*F*(8,36) = 1.358, *P* > 0.05; **Figure 3E**). At 24 h, CCK-8 signals were higher than the untreated control at 5 and 20 ng/mL (*P* < 0.05 for each) and 40 ng/mL (*P* < 0.01), but lower at 320 ng/mL (*P* < 0.001). At 48 h, signals were higher at 10 ng/mL (*P* < 0.05) and at 20 and 40 ng/mL (*P* < 0.001 for each), but lower at 320 ng/mL (*P* < 0.01). Other comparisons were not significant. The response was therefore non-monotonic, and 10, 20 and 40 ng/mL were selected for subsequent functional assays.

rGremlin-1 increased invasion in both SKOV3 and A2780 cells across the tested concentrations (**Figure 3A**). In SKOV3 cells, invasion varied across concentrations (*F*(3,8) = 303.5, *P* < 0.001), and all tested concentrations produced higher invasion than the control (Dunnett-adjusted *P* < 0.001). In A2780 cells, invasion also varied across concentrations (*F*(3,8) = 91.78, *P* < 0.001); increases were significant at 10 ng/mL (adjusted *P* < 0.01) and at 20 and 40 ng/mL (adjusted *P* < 0.001).

Wound closure changed over time differently across concentrations in both SKOV3 and A2780 cells, with significant concentration-by-time interactions in SKOV3 (*F*(5.929,15.81) = 31.96, *P* < 0.001) and A2780 cells (*F*(4.422,11.79) = 19.23, *P* < 0.001; **Figure 3B**). In SKOV3 cells, wound closure was higher than the control at 10 and 40 ng/mL at 24 h (adjusted *P* < 0.05 for each) and at 20 and 40 ng/mL at 48 h (adjusted *P* < 0.05 and *P* < 0.001, respectively). In A2780 cells, wound closure was higher at 20 and 40 ng/mL at 24 h (adjusted *P* < 0.05 for each) and at 10, 20 and 40 ng/mL at 48 h (adjusted *P* < 0.05 for the first two comparisons and *P* < 0.001 for 40 ng/mL). All other prespecified contrasts were not significant.

rGremlin-1 altered EMT-marker abundance in both SKOV3 and A2780 cells (**Figure 3C**). In SKOV3 cells, E-cadherin was lower at 10 ng/mL (*P* < 0.05) and at 20 and 40 ng/mL (*P* < 0.001), whereas N-cadherin was higher at 10 ng/mL (*P* < 0.05) and at 20 and 40 ng/mL (*P* < 0.001). Vimentin was higher at all three concentrations (*P* < 0.001). In A2780 cells, E-cadherin was lower at 10 ng/mL (*P* < 0.01) and at 20 and 40 ng/mL (*P* < 0.001), whereas N-cadherin was higher at all three concentrations (*P* < 0.001). A2780 Vimentin levels differed overall among groups (exact Kruskal-Wallis *P* < 0.001), with a dose-dependent numerical increase relative to the normalized control (mean ± SD: 1.62 ± 0.10, 2.04 ± 0.22 and 2.53 ± 0.23 at 10, 20 and 40 ng/mL, i.e., approximately 62%, 104% and 153% above control); however, only the 40-ng/mL contrast remained significant after Dunn’s adjustment (*P* < 0.01), whereas the 10- and 20-ng/mL contrasts did not (adjusted *P* = 0.91 and 0.12, respectively). These findings support EMT-associated marker changes in response to rGremlin-1, with Vimentin responses differing between SKOV3 and A2780 cells.

The apoptotic fraction decreased after rGremlin-1 exposure in a concentration- and cell-line-dependent pattern (**Figure 3D**). In SKOV3 cells, the reduction was not significant at 10 ng/mL but was significant at 20 and 40 ng/mL (Dunnett-adjusted *P* < 0.001 for each). In A2780 cells, apoptosis was lower at 10 ng/mL (*P* < 0.05) and at 20 and 40 ng/mL (*P* < 0.001).

### 3.4 CAF *GREM1* gain- and loss-of-function alters co-culture ovarian cancer cells

**Figure 4:**
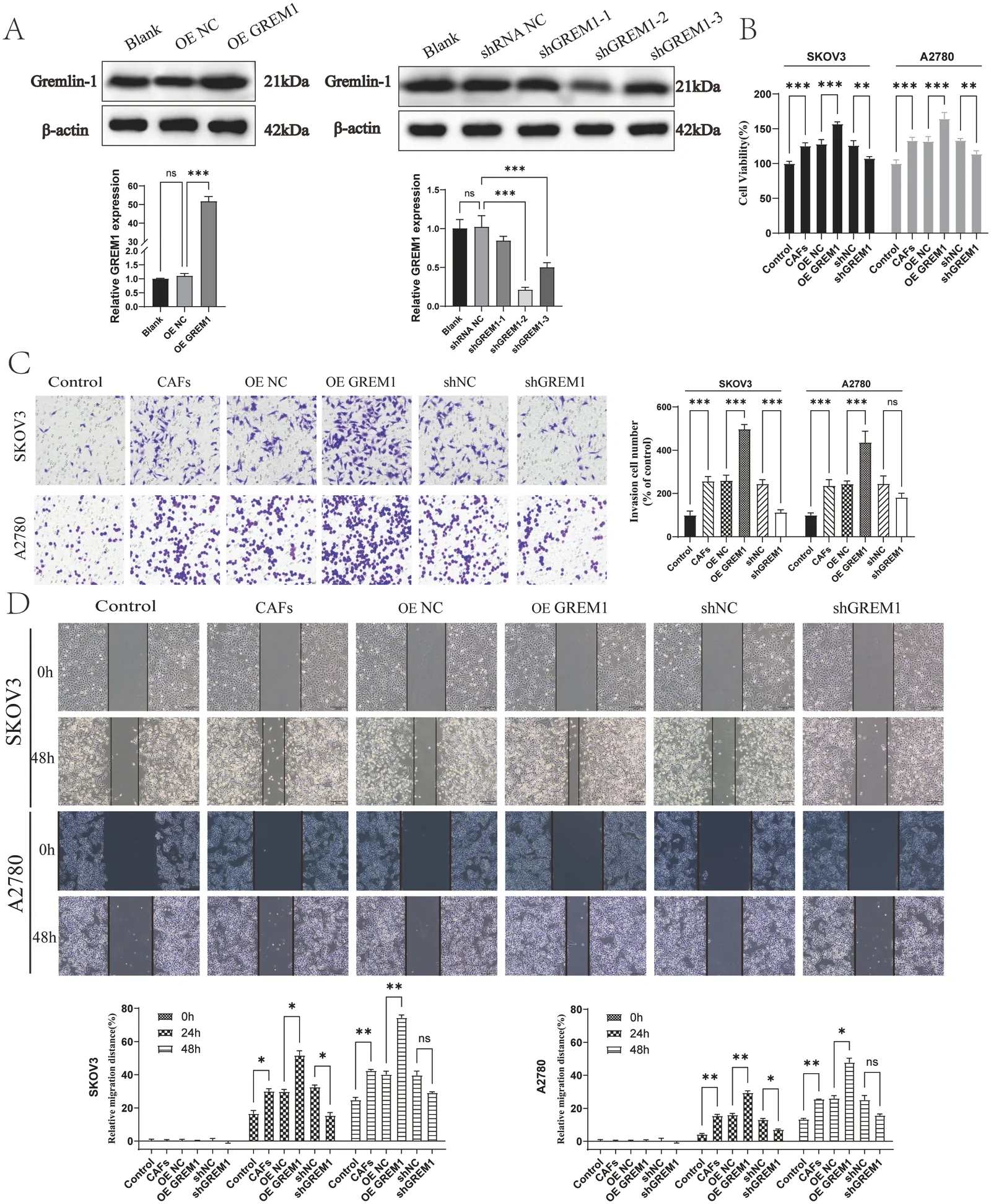
CAF *GREM1* status is associated with ovarian cancer-cell viability, invasion and migration. **(A)** qRT-PCR and western blot analysis of *GREM1* expression in cancer-associated fibroblasts (CAFs) transduced with an overexpression control vector (OE NC), a *GREM1* overexpression vector (OE GREM1), a scrambled shRNA control (shNC) or a *GREM1*-targeting shRNA (shGREM1). For the OE GREM1 qRT-PCR analysis, values were log-transformed before statistical analysis. **(B)** CCK-8-based cell-viability assay of SKOV3 and A2780 cells after 48 h of co-culture under the indicated conditions. **(C)** Transwell invasion assay of SKOV3 and A2780 cells after 24 h of co-culture with CAFs. Representative images are shown at ×200 magnification. **(D)** Wound-healing assay of SKOV3 and A2780 cells after 0, 24 and 48 h of co-culture with CAFs. Representative images at 0 and 48 h are shown at ×200 magnification. Data are presented as mean ± SD (*n* = 3 independent experiments). Panel **(A)** was analyzed using one-way ANOVA with Dunnett-adjusted comparisons. Panels **(B, C)** were analyzed using two-way ANOVA with Tukey-adjusted comparisons. Panel **(D)** was analyzed using repeated-measures two-way ANOVA with Tukey-adjusted comparisons. *P < 0.05, **P < 0.01 and ***P < 0.001; ns, not significant. CAFs, cancer-associated fibroblasts; OC, ovarian cancer; OE GREM1, *GREM1* overexpression; OE NC, overexpression negative control; shGREM1, *GREM1*-targeting shRNA; shNC, scrambled shRNA control.

CAF *GREM1* gain- and loss-of-function models showed the expected directional changes in *GREM1* expression (**Figure 4A**). Relative to OE NC, *GREM1* mRNA abundance was higher in OE GREM1 CAFs (log-transformed analysis; Dunnett-adjusted *P* < 0.001). Among the shRNA constructs, shGREM1–2 produced the largest reduction relative to shNC (Dunnett-adjusted *P* < 0.001) and was selected for downstream experiments. Corresponding western blot measurements showed concordant directional changes. CCK-8-assessed viability differed by co-culture condition (*P* < 0.001) and cell line (*P* < 0.01), with no evidence of a condition-by-cell-line interaction (*P* > 0.05; **Figure 4B**). Relative to monoculture, unmodified CAF co-culture increased the signal in both cell lines (Tukey-adjusted *P* < 0.001 for each). OE GREM1 CAFs produced higher signals than OE NC CAFs in both cell lines (*P* < 0.001 for each), whereas shGREM1 CAFs produced lower signals than shNC CAFs in both cell lines (*P* < 0.01 for each).

Transwell invasion varied with co-culture condition (*P* < 0.001), whereas the overall cell-line effect was not significant (*P* > 0.05); a significant condition-by-cell-line interaction was detected (*P* < 0.01; **Figure 4C**). Relative to monoculture, unmodified CAF co-culture increased invasion in both SKOV3 and A2780 cells (Tukey-adjusted *P* < 0.001 for each). OE GREM1 CAFs produced higher invasion than OE NC CAFs in both cell lines (*P* < 0.001 for each). shGREM1 CAFs reduced invasion relative to shNC CAFs in SKOV3 cells (*P* < 0.001), whereas the corresponding A2780 contrast was not significant. In A2780 cells, shGREM1 CAF co-culture was associated with a numerical reduction in invasion of approximately 25.7% relative to shNC CAF co-culture (mean ± SD: 245.18 ± 35.67 vs 182.06 ± 19.02), although this contrast did not reach statistical significance.

**Figure 5:**
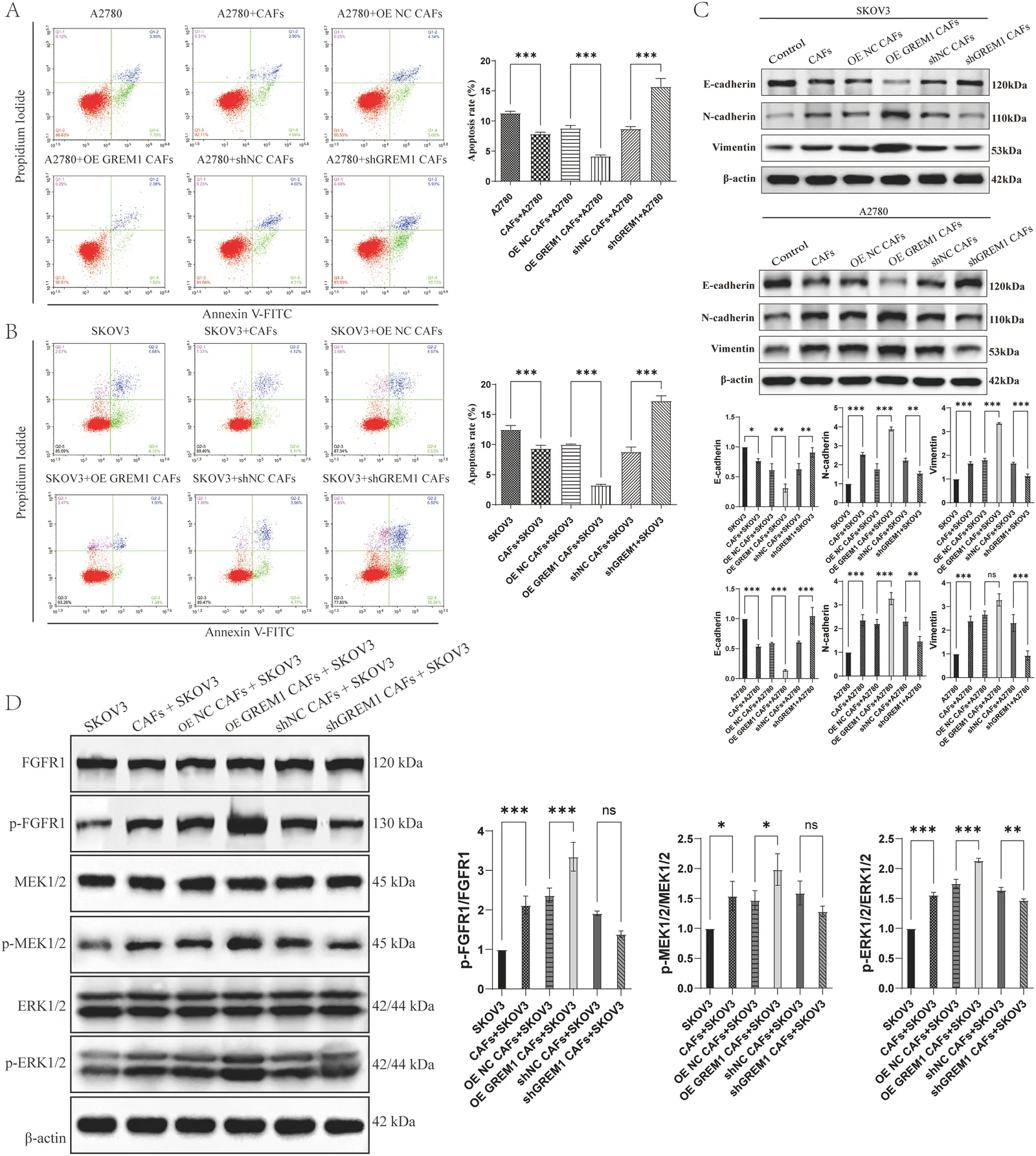
CAF *GREM1* status is associated with apoptosis, EMT-marker changes and FGFR1/MEK/ERK phosphorylation in ovarian cancer cells. **(A)** Flow-cytometric analysis of apoptosis using Annexin V-FITC/PI staining in A2780 cells after 48 h of co-culture with CAFs under the indicated conditions. **(B)** Apoptosis analysis in SKOV3 cells under the same conditions. **(C)** Western blot analysis of E-cadherin, N-cadherin and Vimentin in SKOV3 and A2780 cells after 48 h of co-culture with CAFs. β-actin served as the loading control. For A2780 E-cadherin and Vimentin, values were log-transformed before statistical analysis. **(D)** Western blot analysis of total and phosphorylated FGFR1, MEK1/2 and ERK1/2 in SKOV3 cells after 48 h of co-culture with CAFs. The p-FGFR1/FGFR1, p-MEK1/2/MEK1/2 and p-ERK1/2/ERK1/2 ratios were quantified. β-actin served as the loading control. Data are presented as mean ± SD (*n* = 3 independent experiments). Statistical analyses were performed using one-way ANOVA with Tukey’s multiple-comparisons test. *P < 0.05, **P < 0.01 and ***P < 0.001; ns, not significant. CAFs, cancer-associated fibroblasts; EMT, epithelial-to-mesenchymal transition; FGFR1, fibroblast growth factor receptor 1; MEK, mitogen-activated protein kinase kinase; ERK, extracellular signal-regulated kinase; OC, ovarian cancer.

Wound closure over time differed by co-culture condition in both cell lines, with significant condition-by-time interactions in SKOV3 and A2780 cells (both *P* < 0.001; **Figure 4D**). Relative to monoculture, unmodified CAF co-culture increased wound closure at 24 and 48 h in both cell lines (Tukey-adjusted *P* < 0.05 for each comparison). OE GREM1 CAFs further increased wound closure relative to OE NC CAFs at both time points in both cell lines (*P* < 0.05 for each comparison). shGREM1 CAFs reduced wound closure relative to shNC CAFs at 24 h in both cell lines (*P* < 0.05 for each comparison). At 48 h, wound closure under shGREM1 CAF co-culture remained numerically lower than under shNC CAF co-culture in both cell lines—by 26.7% in SKOV3 (39.81 ± 4.19 vs 29.18 ± 0.83) and 37.4% in A2780 (25.22 ± 4.42 vs 15.79 ± 1.39)—although these contrasts did not reach statistical significance. CAF co-culture reduced apoptosis relative to monoculture in both SKOV3 and A2780 cells (Tukey-adjusted *P* < 0.001 for each; **Figures 5A, B**). OE GREM1 CAF co-culture further reduced apoptosis relative to OE NC CAF co-culture in both cell lines (*P* < 0.001 for each), whereas shGREM1 CAF co-culture increased apoptosis relative to shNC CAF co-culture in both cell lines (*P* < 0.001 for each).

Together with the metabolic, migratory and invasion findings, these gain- and loss-of-function results support an association between CAF *GREM1* status and OC-cell phenotypes under the tested conditions.

### 3.5 CAF *GREM1* status is associated with EMT-marker changes and FGFR1/MEK/ERK phosphorylation in OC cells

To examine molecular correlates of CAF *GREM1*-associated phenotypes, we assessed EMT-marker expression in SKOV3 and A2780 cells and FGFR1/MEK/ERK phosphorylation in SKOV3 cells (**Figures 5C, D**). CAF co-culture reduced E-cadherin and increased N-cadherin and Vimentin in both cell lines (Tukey-adjusted *P* < 0.001 for all comparisons except SKOV3 E-cadherin, *P* < 0.05; **Figure 5C**). OE GREM1 CAFs further reduced E-cadherin and increased N-cadherin in both cell lines (*P* < 0.001 for each); Vimentin increased in SKOV3 cells (*P* < 0.001); in A2780 cells, Vimentin was numerically higher under OE GREM1 than under OE NC CAF co-culture (by approximately 23%; mean ± SD: 3.28 ± 0.26 vs 2.66 ± 0.15), although this contrast was not significant (Tukey-adjusted *P* = 0.30). shGREM1 CAFs increased E-cadherin and decreased N-cadherin and Vimentin in both cell lines (*P* < 0.01 for each).

In SKOV3 cells, CAF co-culture increased the p-FGFR1/FGFR1, p-MEK1/2/MEK1/2, and p-ERK1/2/ERK1/2 ratios relative to monoculture (*P* < 0.05 for each; **Figure 5D**). OE GREM1 CAFs further increased all three ratios relative to OE NC CAFs (*P* < 0.05 for each), whereas shGREM1 CAFs reduced the p-ERK1/2/ERK1/2 ratio relative to shNC CAFs (*P* < 0.01); the other two ratios did not differ significantly. The p-FGFR1/FGFR1 and p-MEK1/2/MEK1/2 ratios showed numerical decreases of 27.3% (mean ± SD: 1.91 ± 0.06 vs 1.39 ± 0.08) and 19.3% (1.59 ± 0.20 vs 1.28 ± 0.09), respectively, after *GREM1* knockdown, without reaching statistical significance. Together, these findings support an association between CAF GREM1 status, EMT-marker changes, and FGFR1/MEK/ERK phosphorylation in the tested models.

## 4 Discussion

Our analyses showed that advanced-stage ovarian cancer was associated with an extracellular-matrix- and stroma-related transcriptional context. The stage IV-versus-stage I comparison identified 113 differentially expressed genes (26), and enrichment analyses converged on extracellular-matrix organization, collagen fibril organization, integrin and syndecan signalling, and related growth-factor pathways (23). These patterns are compatible with literature describing the tumour microenvironment and CAFs as contributors to matrix remodelling, invasion and treatment resistance (10, 27–29). CAF heterogeneity and the dynamic crosstalk with tumor cells provide a plausible context in which individual paracrine factors may have model-dependent effects (30–33). However, enrichment analysis is associative and does not identify a causal stromal driver. Our data do not establish that *GREM1* alone accounts for the advanced-stage transcriptional signature, but they do provide a rationale for examining it as one candidate component of this stromal programme.

*GREM1* was associated with stromal, metastatic and mesenchymal contexts in retrospective ovarian-cancer datasets. Higher expression was observed in tumour stroma, peritoneal metastases, advanced FIGO stages, higher-grade tumours and the mesenchymal subtype, and *GREM1* expression correlated positively with the database EMT score. Retrospective survival analyses using the reported median expression cutoff also associated higher *GREM1* expression with poorer OS or PFS in several cohorts. These observations are consistent with reports linking *GREM1* to stromal remodelling or disease progression in other cancers (34–36). Notably, a recent systematic review of 36 studies delineated dual, context-dependent roles of GREM1 in tumor-stroma interactions: stromal and CAF-derived GREM1 can foster an immunosuppressive microenvironment, extracellular-matrix deposition and therapy resistance in several malignancies, whereas in other settings, such as melanoma, GREM1 restrains invasive behavior (37). Our observation that CAF GREM1 status was associated with pro-migratory and EMT-related changes in ovarian cancer-cell co-culture models is consistent with the pro-tumoral facet of this dual role, while the cell-line dependence we observed echoes its context-dependent nature. They remain associative, however, and may reflect tumour composition, cohort structure or other confounding factors. The independent tissue cohort showed a numerical increase in benign and malignant groups, but the three-group comparison was not significant, and bulk-tissue RNA cannot localize *GREM1* to CAFs. Thus, the present data support *GREM1* as a candidate stromal-associated factor for further study.

The recombinant-protein and co-culture experiments provide complementary evidence. rGremlin-1 exposure altered invasion, wound closure, EMT-marker abundance and apoptosis in SKOV3 and A2780 cells. In parallel, CAF co-culture and CAF *GREM1* gain- and loss-of-function were associated with changes in CCK-8-assessed viability, invasion, migration and apoptosis. These findings support a contribution of CAF *GREM1* status to selected OC-cell phenotypes under the tested conditions.

The co-culture experiments also revealed cell-line and time dependence. *GREM1* knockdown reduced invasion in SKOV3 cells, whereas the corresponding A2780 invasion contrast was not significant; migration and apoptosis responses were more consistent across the two lines, but the 48 h migration contrasts after knockdown were not significant. This pattern argues against treating the two cell lines as interchangeable and is compatible with compensatory paracrine signals or differences in pathway wiring. Such divergence is compatible with the context-dependent, dual behavior of GREM1 documented across tumor types (37). In that framework, stromal GREM1 can drive pro-malignant phenotypes, but its effects can be modified by the cellular context or the presence of alternative paracrine signals, such as TGF-β (31). This cell-line-specific response to CAF-derived Gremlin-1 highlights the complexity of the TME and indicates that the molecular mechanisms of CAF-induced invasion are not universal across all OC cell types.

EMT-marker changes were observed after rGremlin-1 exposure and in CAF co-culture, but the marker pattern was not identical across cell lines. In SKOV3 cells, CAF *GREM1* status coincided with changes in p-FGFR1/FGFR1, p-MEK1/2/MEK1/2 and p-ERK1/2/ERK1/2 ratios. The loss-of-function comparison reduced ERK1/2 phosphorylation, whereas the p-FGFR1 and p-MEK1/2 contrasts were not significant. These data identify FGFR1/MEK/ERK phosphorylation as a candidate correlate of the observed SKOV3 phenotype. Because no inhibitor, genetic perturbation or rescue experiment was performed, pathway necessity, sufficiency and direct ligand–receptor engagement remain untested. The absence of recombinant-protein specificity controls and the use of commercially supplied fibroblasts marketed as CAFs further limit mechanistic interpretation.

The scope of these interpretations is limited by the model, cohort and pathway-validation constraints detailed in Section 5.

## 5 Limitations and future directions

Several limitations of this study should be acknowledged. First, the fibroblasts were commercially supplied as CAFs but were not comprehensively re-characterized in our laboratory. Limited Vimentin immunofluorescence showed that more than 90% of the cells were Vimentin-positive, supporting fibroblast identity (**Supplementary Figure S1**). Intracellular *GREM1* mRNA and Gremlin-1 protein expression were measured by qRT-PCR and western blotting to validate the gain- and loss-of-function models (**Figure 4A**). However, Vimentin is not CAF-specific, comprehensive CAF-marker profiling was not performed, and secreted extracellular Gremlin-1 was not quantified. Therefore, the identity and heterogeneity of the stromal model, as well as the extent to which the observed effects reflect CAF-derived Gremlin-1, remain incompletely defined. Future studies should use independently validated primary CAFs, perform comprehensive CAF-marker profiling and quantify secreted Gremlin-1.

Second, the tissue cohort was small (normal, *n* = 4; benign, *n* = 7; malignant, *n* = 8), and measurements were performed on bulk-tissue homogenates. An exploratory IHC pilot (**Supplementary Figure S2**) included one mucinous and one serous ovarian-cancer specimen; a colon-tissue section was included as a reference for the manufacturer-reported positive-control tissue type. Heterogeneous DAB immunoreactivity was observed, but the pilot was qualitative and lacked standardized scoring and CAF-marker co-localization. It therefore cannot establish histotype-specific abundance or assign Gremlin-1 staining specifically to CAFs. The nonsignificant three-group comparison and the absence of microdissection or CAF isolation further limit inference about stromal-specific *GREM1* expression. The CSIOVDB and KM Plotter analyses were retrospective and associative; they do not establish *GREM1* as an independent or clinically validated prognostic biomarker. Larger, independent cohorts with cell-type-resolved assays and multivariable analyses are needed.

Third, the cell-based experiments used three independent biological experiments and two OC cell lines, limiting statistical precision and generalizability. With three replicates per group, statistical power is limited, and non-significant comparisons—such as the A2780 invasion contrast after GREM1 knockdown and the p-FGFR1/FGFR1 and p-MEK1/2/MEK1/2 contrasts after knockdown—may reflect Type II error rather than a true absence of effect. The findings should therefore not be extrapolated directly to primary tumors, organoids or *in vivo* disease. The recombinant-protein experiments lacked neutralizing-antibody, heat-denatured-protein and matched-protein controls. Although the manufacturer reported a purity of ≥85% and endotoxin levels below 1 EU/µg, ligand-specific effects were not established and should be interpreted cautiously.

Finally, FGFR1/MEK/ERK phosphorylation was evaluated only in SKOV3 cells, and no pharmacological inhibition, genetic perturbation or rescue experiments were performed. This pathway should therefore be regarded as a candidate correlate rather than a proven mediator or required effector of the observed phenotypes. Future studies should test pathway dependence in additional models and *in vivo* systems, together with stromal-resolved analyses and clinically annotated cohorts.

## 6 Conclusion

Taken together, our study found that CAF *GREM1* status was associated with changes in ovarian cancer-cell viability, migration, invasion, apoptosis and EMT-related markers in SKOV3 and A2780 co-culture models. In SKOV3 cells, these changes coincided with altered FGFR1/MEK/ERK phosphorylation, supporting this pathway as a candidate correlate rather than a proven mediator. Further studies are needed to determine the clinical relevance of stromal Gremlin-1 and whether the observed phenotypes depend on FGFR1/MEK/ERK signaling.

## Data Availability

The datasets presented in this study can be found in online repositories. The names of the repository/repositories and accession number(s) can be found in the article/**Supplementary Material**.
