# Supplementary material for: Fibroblast-derived gremlin-1 Is associated with ovarian cancer-cell phenotypes in co-culture models
Source: Front Oncol. 2026 Sep 7;16:1906132. doi: 10.3389/fonc.2026.1906132 (PMC13590338; doi:10.3389/fonc.2026.1906132)
Supplement: Supplementary file 1 [file SupplementaryFile1.docx]

**100X-DAPI	100X-Vimentin	100X-Fluorescence**

2**00X-DAPI	200X-Vimentin	200X-Fluorescence**

**Supplementary Figure S1. Vimentin immunofluorescence of commercially supplied fibroblasts marketed as cancer-associated fibroblasts (CAFs).**

Immunofluorescence staining for Vimentin (green) was performed, with nuclei counterstained with DAPI (blue). Representative images were acquired at ×100 and ×200 magnification. Scale bars, 100 μm. More than 90% of the cells were Vimentin-positive, supporting fibroblast identity. The cells were purchased from iCell Bioscience Inc. (Shanghai, China) and authenticated by the supplier. Vimentin staining alone does not establish a complete CAF phenotype.
