# Supplementary material for: Fibroblast-derived gremlin-1 Is associated with ovarian cancer-cell phenotypes in co-culture models
Source: Front Oncol. 2026 Sep 7;16:1906132. doi: 10.3389/fonc.2026.1906132 (PMC13590338; doi:10.3389/fonc.2026.1906132)
Supplement: Supplementary file 2 [file SupplementaryFile2.docx]

**Supplementary Figure S2. Exploratory Gremlin-1 immunohistochemistry in ovarian-cancer tissue.** (A) Mucinous ovarian-cancer tissue. (B) Serous ovarian-cancer tissue. (C) Colon tissue included as a reference for the manufacturer-reported positive-control tissue type. Brown diaminobenzidine (DAB) staining was observed in the sections, but staining intensity and distribution were heterogeneous.

This pilot immunohistochemistry (IHC) experiment was performed to assess the feasibility of detecting Gremlin-1 in ovarian-cancer tissue. The experiment included one mucinous ovarian-cancer specimen, one serous ovarian-cancer specimen and a colon-tissue section used as a reference for the manufacturer-reported positive-control tissue type. Because only one specimen was examined for each ovarian-cancer histotype, these images are exploratory and were not used for statistical comparison or biological validation.

**Antibody and assay information**

| **Item** | **Information** |
| --- | --- |
| Antibody | Anti-Gremlin 1 antibody |
| Product | Abcam ab22138 |
| Host and clonality | Rabbit polyclonal IgG |
| Species reactivity | Human |
| Application | IHC-P; assay-dependent dilution |
| Purification | Protein G purified |
| Manufacturer-reported positive control | Human colon cancer tissue |
| Cellular localization reported by manufacturer | Secreted |
